# Subclinical hypothyroidism: Is it important in intracytoplasmic sperm injection cycles?

**DOI:** 10.4274/tjod.48108

**Published:** 2017-06-15

**Authors:** Eray Çalışkan, Rahime Nida Ergin, Deniz Can Öztekin, Bülent Kars, Seda Çakır, Kenan Sofuoğlu

**Affiliations:** 1 Bahçeşehir University Faculty of Medicine, Department of Obstetrics and Gynecology, İstanbul, Turkey; 2 Ege Maternity and Gynecology Training and Research Hospital, Clinic of Obstetrics and Gynecology, İzmir, Turkey; 3 University of Health Sciences, Kartal Dr. Lütfi Kırdar Training and Research Hospital, Clinic of Obstetrics and Gynecology, İstanbul, Turkey; 4 University of Health Sciences, Zeynep Kamil Maternity and Children’s Health Training and Research Hospital, Clinic of Obstetrics and Gynecology, İstanbul, Turkey

**Keywords:** female, infertility, intracytoplasmic sperm injection, Subclinical hypothyroidism, pregnancy rate

## Abstract

**Objective:**

To compare intracytoplasmic sperm injection (ICSI) outcomes of women with subclinical hypothyroidism with those of euthyroid women.

**Materials and Methods:**

A retrospective case-control study was conducted. Out of 2529 ICSI cycles evaluated, 41 women with hypothyroidism, 28 women with hyperthyroidism, and 128 women with subclinical hyperthyroidism were excluded, and 2336 cycles were analyzed. Women were identified as having subclinical hypothyroidism (case group, n=105) in the presence of a thyroid-stimulating hormone level >4.5 mU/L and normal free T4 and compared with euthyroid controls (n=2231).

**Results:**

The mean age, body mass index, day 3 follicle-stimulating hormone level, and antral follicle count of the study patients were similar to the control group (p>0.5). The cycle cancellation rate of the study group was similar to the control group (13.3% vs. 7.6%, p=0.1). The clinical pregnancy rate was 21.2% in the study group, which was significantly lower than the 35.8% in the control group (p=0.04). The take-home baby rate was also significantly lower in the study group compared with the control groups (13.5% vs. 31.4% respectively, p=0.01).

**Conclusion:**

Both the clinical pregnancy rate and the take-home baby rate is lower in women with subclinical hypothyroidism at the time of ICSI cycle.

## PRECIS:

In this multi-centered retrospective case-control study, we aimed to determine intracytoplasmic sperm injection (ICSI) outcomes of infertile women with subclinical hypothyroidism versus euthyroid infertile women, including higher numbers of ICSI cycles.

## INTRODUCTION

Various experimental cell culture studies involving human and some animal species have shown that thyroid hormones have, to some degree, a stimulatory effect on granulosa and/or thecal cells^(1,2,3)^. Therefore, it is anticipated that disturbances in this thyroid-ovarian interaction might exert a negative influence on pregnancy as previously shown^(4,5)^. In this manner, hypo- or hyper-thyroidism have their own ways of management; however, it is not clear for subclinical hypothyroidism, which is defined as a serum thyroid-stimulating hormone (TSH) above the defined upper limit of the reference range, with a serum free thyroxine (fT4) within the reference range^(6)^. It is rather a laboratory diagnosis because patients with subclinical hypothyroidism have no or fewer symptoms^(6)^. The routine serum assays used to rule out subclinical hypothyroidism are not recommended, except in some specific patient groups including pregnant women due to possible developmental problems of the fetus related to maternal high TSH levels^(6)^. However, a recent Cochrane database systemic review concluded that there was insufficient evidence to recommend the use of one intervention for clinical or subclinical hypothyroidism before or during pregnancy over another, for improving maternal, fetal, neonatal, and childhood outcomes^(7)^.

In a recent study of limited numbers of pregnant women with subclinical hypothyroidism, T4 treatment was reported to result in similar clinical pregnancy rates per cycle but higher embryo implantation rates, live birth rates, and lower miscarriage rates compared with the no treatment group^(8)^. In contrast with this study, subclinical hypothyroidism and overt hypothyroidism was shown not to benefit from T4 treatment in terms of the clinical pregnancy rates per started cycle, implantation rates, and the live birth rates per started cycle compared with euthyroid controls^(9)^. Therefore, in this multi-centered retrospective case-control study, we aimed to determine intracytoplasmic sperm injection (ICSI) outcomes of infertile women with subclinical hypothyroidism versus euthyroid infertile women, including higher numbers of ICSI cycles.

## MATERIALS AND METHODS

Hospital records of infertile women who attended participant referral study centers of assisted reproduction therapy (ART) between 2014 and 2015 were analyzed retrospectively in this retrospective case-control study. Patients without laboratory studies of thyroid functions, those with missing data related to ART, and those with thyroid function abnormalities such as thyroid autoimmunity diseases other than subclinical hypothyroidism were not included in the study. Ovarian stimulations were performed using a combination of gonadotropin-releasing hormone agonist/antagonist and follicle-stimulating hormone (FSH)/human menopausal gonadotropin. When the leading follicle diameter was larger than 17 mm, human chorionic gonadotropin (hCG) was given. Oocytes were retrieved 35 h after hCG administration. Following oocyte preparation, insemination was performed using ICSI in all cases one hour later. After insemination, eggs were individually cultured until day 5 for blastocyst transfer.

Data related to ICSI cycles, patients’ demographics, laboratory results, and pregnancy outcomes were compared between the two groups of infertile women; the study group included infertile women with subclinical hypothyroidism and the control group comprised women who were euthyroid. Women were identified as having subclinical hypothyroidism in the presence of a TSH level >4.5 mU/L and a serum fT4 within the reference range^(6)^. The patients with hyperprolactinemia had been treated with levothyroxine. Hyperprolactinemia is also common in subclinical hypothyroidism^(10)^; therefore, patients with both subclinical hypothyroidism and hyperprolactinemia were included in the study and were treated with levothyroxine and a dopamine agonist.

### Statistical Analysis

Out of 2529 ICSI cycles with eligible data, 41 women with hypothyroidism, 28 women with hyperthyroidism, and 128 women with subclinical hyperthyroidism were excluded, and 2336 cycles were analyzed. The related statistical comparisons of groups were performed using the with ANOVA test and chi-square test, where appropriate. Correlation analyses were performed with Pearson’s correlation. Statistical analyses were performed using SPSS statistics software (SPSS Statistics for Windows, version 17.0; SPSS Inc., Chicago, USA). The p value was set as <0.05 for significance.

## RESULTS

Of the included 2336 cycles, 105 women were identified as having subclinical hypothyroidism (study group) and 2231 women were included in the euthyroid control group. The mean age, body mass index, type and duration of infertility were similar between the groups; only causes of infertility differed between the groups (Table 1). Both the study group and control euthyroid group were statistically indifferent considering infertility analysis values including FSH, luteinizing hormone (LH), estrogen, total antral follicle count, and sperm analyses (Table 2). Of the cases with subclinical hypothyroidism, 46% (n=48) had associated hyperprolactinemia. The rate of patients with TSH values equal or higher than 10 was 11% (n=12). With the exceptions of significantly lower estradiol measurements and endometrial thickness on hCG day, the outcomes of controlled ovarian hyper-stimulation and ICSI of the study group were similar to those of the control group (Table 3). The cycle cancellation rate of the study group was similar to the control group (13.3% vs. 7.6%, p=0.1). The clinical pregnancy rate was 21.2% in the case group, which was significantly lower than the 35.8% in the control group (p=0.04). The take-home baby rate was also significantly lower in the study group compared with the control group (13.5% vs. 31.4%, respectively; p=0.01) (Table 4). Miscarriage rates were also higher in the study group compared with the control group (36% vs. 24%); however, the result was not statistically significant.

When the outcomes of patients with subclinical hypothyroid with and without hyperprolactinemia were compared in terms ICSI cycle parameters and pregnancy outcomes, none was found as significantly different (Table 5). In the subgroup analysis of patients with subclinical hypothyroidism according to TSH level (<10 versus TSH ≥10) for the outcomes of ICSI and pregnancy outcomes, only significantly lower levels of estradiol on hCG day and a significantly higher rate of cycle cancellation were present in patients with TSH ≥10 (Table 6).

## DISCUSSION

Experimental studies have demonstrated an interaction of thyroid hormones with ovarian function, and clinical studies have also reported negative effects of thyroid hormonal excess or defects with overt clinical signs and symptoms^(1,2,3,4,5)^. However, diagnosis of subclinical hypothyroidism depends on laboratory analysis rather than clinical symptoms; therefore, its possible effects on fertility status and pregnancy rates with ART need to be clarified. To achieve this aim, previous studies reported contrary results including improved pregnancy rates with T4 treatment or no change in pregnancy rates with T4 treatment, even in infertile patients with overt hypothyroidism^(8,9,11,12,13,14)^. In this multicenter retrospective study, the TSH cut-off value was set as 4.5 mIU/L for the diagnosis of subclinical hypothyroidism, as suggested previously by the consensus of the American Endocrine Society, the American Thyroid Association, and the American Association of Clinical Endocrinologists^(6)^.

However, in a relatively recent study, two different cut-off values (2.5 mIU/L vs. 4.5 mIU/L) values were compared in terms of rates of clinical pregnancy, delivery or miscarriage in a large, retrospective cohort study of patients undergoing their first *in vitro* fertilization (IVF) cycle^(11)^. No statistical differences were found between the groups and it was suggested that lowering the TSH cut-off value would increase the diagnosis rate of subclinical hypothyroidism five-fold^(11)^. In our study, however, when we compared groups with TSH values of 4.5 to 9.9 or ≥10 mIU/L, we found a significant higher rate of cycle cancellations in patients with TSH ≥10 mIU/L. Nevertheless, the clinical pregnancy rates and take-home baby rate did not differ, in a similar manner with the previous study, which compared TSH values of 2.5 vs. 4.5 mIU/L^(11)^. In this present study, we used an ICSI population to determine the possible effects of subclinical hypothyroidism. Previously, it was shown that overt hypothyroidism was related with a decreased chance of achieving pregnancy following IVF, even with appropriate treatment^(5)^. In another study that determined outcomes of controlled ovarian hyper-stimulation in women with thyroid autoimmune disease, oocyte pickup and embryo transfer, the performance of recombinant-FSH was significantly poorer in patients with thyroid autoimmune disease^(12)^. When TSH values with a cut-off value of 2.5 IU/L were compared, significantly higher serum E2 concentrations was determined in those with <2.5 IU/L^(12)^. In our study, we found a similar significant difference when comparing TSH values of 4.5-9.9 vs. 10 or more IU/L. In our study, the rate of subclinical hypothyroidism was 5%; however, in a previous study, it was reported as high as 13.9%, which was a significantly higher rate compared with fertile patients (3.9%)^(13)^. In the present study, the euthyroid group had an incidental higher frequency of endometriosis, anovulation, and unexplained infertility, all of which are expected to decrease success rates in ICSI cycles by themselves. However, despite this, we found a significantly lower clinical pregnancy and take-home baby rate in the study group compared with the control group. 

The findings of the current study and some other previous observations as mentioned above need to be verified with further epidemiologic and experimental studies. In a study that compared laboratory results of blood samples drawn every 10 minutes during a 24-h period for pulse analysis of LH, TSH, and prolactin, no difference was found between euthyroid hypothyroid patients or those with subclinical hypothyroidism^(14)^. It was concluded that corpus luteum insufficiency in female infertility could not be explained by subclinical hypothyroidism and thus should not be treated with L-thyroxin for fertility reasons^(14)^. However, another study concerning hypothyroidism in IVF cycles concluded that high circulating estradiol during superovulation for IVF increased the binding of thyroxin to thyroxin-binding globulin, resulting in relative hypothyroidism during a super-ovulation cycle in women taking thyroxin replacement therapy^(15)^.

### Study Limitations

Major limitation of this study is that it depends on retrospective data analyses, which necessitates to be confirmed by further well designed prospective clinical study regarding effects of subclinical hypothyroidism on ICSI cycles.

## CONCLUSION

In conclusion, both the clinical pregnancy and take-home baby rate are lower in women with subclinical hypothyroidism at the time of ICSI cycle, regardless of T4 treatment.

## Figures and Tables

**Table 1:**
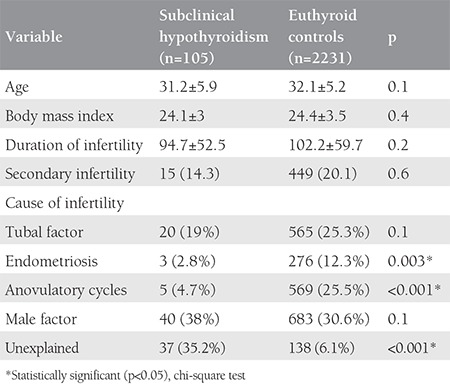
Demographics of the study population and causes of infertility

**Table 2:**
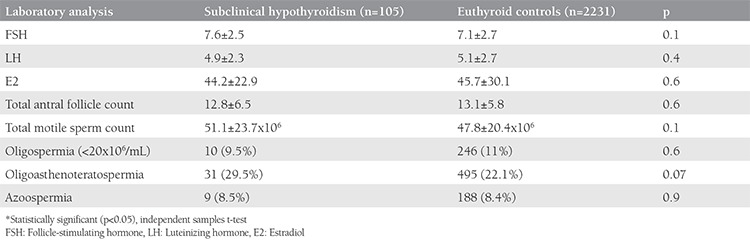
Laboratory analyses of the women and men

**Table 3:**
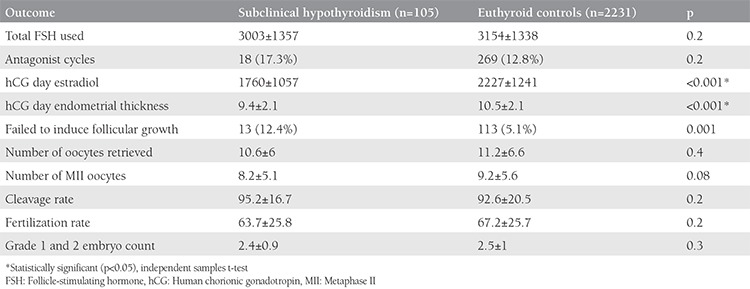
Outcome of controlled ovarian hyperstimulation and intracytoplasmic sperm injection

**Table 4:**
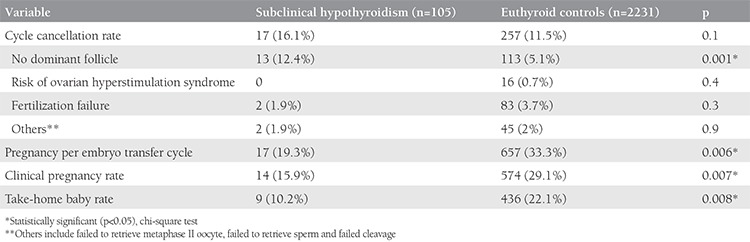
Cycle cancellation and pregnancy outcome

**Table 5:**
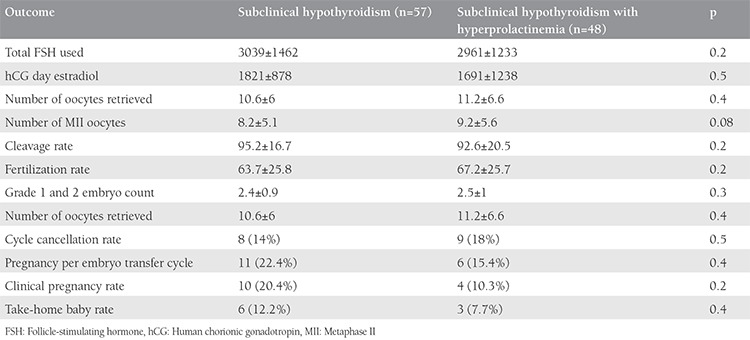
Outcome of patients with subclinical hypothyroidism with and without hyperprolactinemia (>30)

**Table 6:**
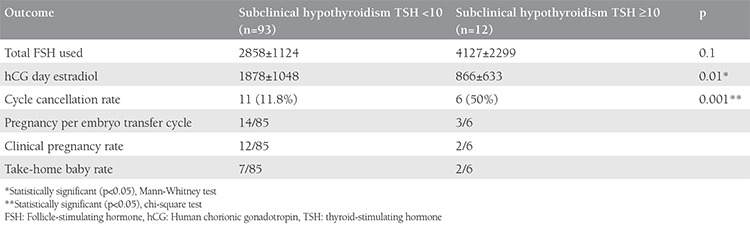
Intracytoplasmic sperm injection outcomes according to thyroid-stimulating hormone values in patients with subclinical hypothyroidism
